# Supplementary material for: Development and validation of a multigene variant profiling assay to guide targeted and immuno therapy selection in solid tumors
Source: PLoS One. 2021 Feb 8;16(2):e0246048. doi: 10.1371/journal.pone.0246048 (PMC7870065; doi:10.1371/journal.pone.0246048)
Supplement: Attachment — Submitted filename: CellDx_PLOSONE_review (1).docx [file pone.0246048.s009.docx]

The authors present a multigene variant profiling assay (CellDx) and validate its high sensitivity using known variants in reference samples. However, the specificity and accuracy of this assay need further validation. The definition of true negative variants needs to be clarified. Furthermore, the oncogenicity of the detected variants and whether they are biologically actionable are annotated by external databases in this manuscript. CellDx alone is not sufficient to provide actionable indications for targeted therapy. In addition, whether and how the detected variants could be used as biomarkers to stratify patients also needs to be elaborated. More evidence is needed to support the clinical significance of CellDx.

| **Manuscript-related** | |
| --- | --- |
| **Excerptions** | **Comments** |
| Page 2, paragraph 1: “CellDx includes Next Generation Sequencing (NGS) profiling of gene 29 variants in a targeted 452-gene panel…” | 1. Please clarify the reason why selecting these 452 genes. What is their particularity? Are they known cancer genes? |
| Page 4, paragraph 2: “Analytical validation was performed on 116 reference samples including Formalin Fixed Paraffin Embedded (FFPE) tumor tissue, tumor DNA or tumor RNA (Supplementary Tables S1) which were obtained from various sources such as College of American Pathologist (CAP), European Molecular Genetics Quality Network (EMQN), Coriell Institute of Medical Research (CIMR) as well as various commercial providers.” | 2. Have the genetic alterations in the 116 reference samples been orthogonally validated? If the mutation set in these samples serve as ground truth and thus are used for evaluating the sensitivity, specificity and accuracy of CellDx, are they identified by orthogonal assays? How do you get the benchmark mutation set? Please clarify. |
| Minor | 3. The name of column 1 and 2 in supplementary table 1 are the same. |
| Page 7, paragraph 3: “Tumor mutational burden (TMB) was determined in 133 FFPE/fresh tissue samples by NGS using ion Ampliseq 452 gene panel.” | 4. There are more than 133 samples involved in this study. When studying tumor mutation burden, why do you only focus on these 133 samples? Please clarify. |
| Page 7, paragraph 3: “TMB was defined as the total number of somatic mutations and was calculated by counting non-synonymous, coding somatic variants as well as pathogenic germline variants in tumor genome across ~2.02 MB in 452 genes.” | 5. Tumor mutational burden (TMB) is defined as the total number of somatic mutations and pathogenic germline variants in 452 genes.    a. What are defined as pathogenic germline variants and where do you get the benchmark dataset? Is their pathogenicity specific for cancer? In some widely used datasets such as ClinVar, the mutations are responsible for different diseases. Please clarify.    b. The frequency of germline and somatic variants are not the same, and the two class of variants may also have distinct functional impacts in cancer. It is not fair to directly add the mutation counts.    c. The background mutation rates of the 452 genes could be diverse. The signal may be diluted if the mutation burden is unusually high in one or a small fraction of genes but remains the same in the other genes. Also, why is the overall mutation burden in these 452 genes informative? What is the particularity of this gene panel? Please clarify. |
| Page 8, paragraph 1: “TMB levels was divided into three groups Low (1 - 5 mut/MB), Intermediate (6 - 10 mut/MB) and High (>10 mut/MB)” | 6. What is the basis of the arbitrary cutoff? Do you take into consideration the background mutation rate when setting the cutoff? What is the expected mutation burden? Please clarify. |
| Page 9, paragraph 3: “A total 51 samples were used to calculate the sensitivity of CellDx test…”  Page 10, paragraph 2: “The analytical specificity of the CellDx assay was assessed for SNA, CNAs, large indels, small indels, gene fusions, MSI, PD-l and MMR, on 59 reference samples…” | 7. The two sample sets for assessing sensitivity and specificity are mutual exclusive. Why? Although there are some problems with the calculation of specificity (Please see Comment 10), the samples in both sets could be used to assess sensitivity. Why not merge the two sets and assess the overall sensitivity so that the result would be more comprehensive? |
| Page 10, paragraph 2: “Additionally, 20 known negative samples for PDLI (10 for 28-8, 10 for 22C3), 8 known negatives for MMR and 2 known negative for MSI samples were included for specificity… The analytical specificity was obtained as the fraction of true negatives (TN) among the sum total of TN and false positive (FP), [TN / (TN + FP)].” | 8. As indicated in the manuscript, the specificity is calculated as TN / (TN + FP). How do you get the value of TN? Is it defined as the number of known negative samples where no genetic alteration is detected? If so, the unit of TN and FP will be different. The unit of FP is the number of mutations, while the unit of TN is the number of samples. Therefore, this may not be an appropriate way to assess specificity. |
| Page 10, paragraph 3: “Accuracy was defined as the proportion (%) of TP and TN among the sum total of TP, TN, FP and FN.” | 9. The same problem as described in Comment 10. The unit of TN is different from that of TP, FP and FN. In this case it may not be an appropriate way to assess the accuracy by calculating (TP + TN) / (TP + TN + FP + FN). |
| Page 11, paragraph 1: “Reproducibility was assessed for Ampliseq 452 gene panel and PD-L1 status, whereas Repeatability was assessed for Ampliseq 452 gene panel and MSI status.” | 10. Why not assess reproducibility and repeatability for 452 gene panel, MSI, PDB-L1 and MMR respectively? Please clarify the reason why assessing reproducibility for gene panel and PD-L1 while assessing repeatability for gene panel and MSI. |
| Page 11, paragraph 2: “The real-world performance of the CellDx assay estimated by analysing clinical samples for detection of actionable features in DNA/RNA, as well as in determining status of PD-L1, MMR, MSI and TMB.” | 11. The statement here is confusing. How do you estimate the clinical performance? Do you mean analyzing clinical samples using CellDx and looking into the genetic alterations detected, and see how many of them are biologically actionable? Please provide more details. |
| Page 12, paragraph 2: “Among the 27 samples which were analyzed by NGS, 176 variants were detected in 65 genes including 148 SNA, 17 indels, 1 CNA and 10 gene fusions. There were 2 false negatives (FN) for which BAM files of the samples were reviewed using integrated genome viewer: one FN was due to 2% variant allele frequency (VAF) of EGFR.pL858R and one FN was due to 2.8 copies of MET. The overall analytical sensitivity for SNA, small indels, large indels, CNA and gene fusion were 99.99%...The overall sensitivity of the CellDx assay was 99.99%.” | 12. As indicated here, for NGS, TP = 176 and FN = 2. The analytical sensitivity should be calculated as TP / (TP + FN) = 98.9%. How was 99.99% calculated? In Supplementary Table S5, I found no false negative listed there, which is in conflict with the statement of 2 FN. Furthermore, in the “Sensitivity” section of Table S5, the first entry is “NGS: NGS: CNA” and the fifth entry is “NGS: CNA”, which seems to be duplicate. The TP value in the “Overall” entry is 51, how was 51 calculated? Why is the overall sensitivity of CellDx 99.99%? Please clarify. |
| Page 13, paragraph 1: “…1078 true negative (TN) variants were correctly detected.” | 13. How are the TN variants defined? If they are not known variants and are not detected by NGS, how do you get the variant list? Also, I couldn’t get this number “1078” by summing the NGS entries in the “Specificity” section in Supplementary Table S5. In this table section, again, what do “NGS: NGS: CNA” and “Overall” mean? Please clarify. |
| Page 13, paragraph 2: “Accuracy of NGS based on detection of all TP variants and 2 FP among all TN variants was 99.9%. Accuracies for PD-L1, MMR and MSI were also be 99.99%. The overall accuracy of the CellDx assay was 99.32% (95% CI: 98.71% - 99.69%).” | 14. As the questions remain to be answered on the calculation of sensitivity and specificity, the validity of this accuracy is unclear. |
| Page 13, paragraph 3: “Repeatability was determined for NGS and MSI…Reproducibility was determined for the NGS and PD-L1.” | 15. Why do you only assess the repeatability of NGS and MSI? Why not assess the repeatability of MMR and PD-L1? 16. Why only assess the reproducibility of NGS and PD-L1? Why not assess the reproducibility of MSI and MMR? 17. What is the definition of precision? How do you integrate repeatability and reproducibility to get the precision value?   Please clarify. |
| Page 14, paragraph 2: “To evaluate the LOD of variant calling, 2 FFPE tissue samples were analysed;” | 18. It seems that the LOD assessed from 2 samples may not be solid enough. |
| Page 15, paragraph 2: “Pathogenic and likely pathogenic somatic variants as well as somatic variants of unknown significance (VUS) were detected in 96.99% (129/133) of samples which were analyzed by NGS.” | 19. What is the source of pathogenic and likely pathogenic somatic variants? How did you get the benchmark dataset? Are these variants causal in cancer? 20. Where do you get the VUS list? The function of VUS is unknown. By stating that these VUS together with pathogenic variants were detected in 96.99% samples, what conclusion do you want to make? |
| Page 15, paragraph 2: “We analyzed the clinical associations of all detected variants in all patients by mapping driver mutations to clinical annotation databases (CIViC and OncoKB). Among the 133 patients’ samples, 852 unique mutations were detected, of which 784 were oncogenic and 47 were actionable. ” | 21. All detected variants are called “driver mutations” here, which is not rigorous. How do you know they are all oncogenic? 22. The oncogenicity of the detected variants is annotated by external databases. CellDx itself is not able to identify either oncogenic or actionable genetic alterations. |
| Page 15, paragraph 2: “Among the 128 patients, 31 patients (27%) had low TMB, 50 patients (43%) had an intermediate TMB and 34 patients (30%) had high TMB…Among the 112 samples evaluated by IHC for PD-L1 expression, 22 samples (19.64%) were positive (TPS score >1%) for PD-359 L1. Among the 105 samples evaluated for MMR status, LNE: MSH2 + MSH6 was observed in 1 sample. Among the 138 samples evaluated for MSI status, 6 samples (4%) returned positive findings with BAT-26 (5%), NR-21 (2%), NR-24(2%) and MONO-27 (1%).” | 23. How do TMB, PD-L1 expression, MMR status, and MSI status stratify patients? What does it mean if a patient has low, intermediate or high TMB? What does it mean if a sample is positive for PD-L1 or MSI? Does that mean worse clinical outcome? What is the clinical significance of studying TMB, PD-L1 expression, MMR status, and MSI status? Please clarify. |
