# Supplementary material for: Development and validation of a multigene variant profiling assay to guide targeted and immuno therapy selection in solid tumors
Source: PLoS One. 2021 Feb 8;16(2):e0246048. doi: 10.1371/journal.pone.0246048 (PMC7870065; doi:10.1371/journal.pone.0246048)
Supplement: Attachment — Submitted filename: Response to Reviewers.docx [file pone.0246048.s011.docx]

***QUERY 1: In Supplementary Table S7 of the revision, for NGS:SNA, the numbers for TP, FP, TN and FN are 148, 9, 1053 and 1, respectively. However, in the Supplementary Table S5 of the previous version, the number for FN was 0. Please clarify what re-analyses have led to this big change in FN, although the absolute number being only 1.***

**RESPONSE**: In the version submitted initially, Sensitivity and Specificity were evaluated in separate sample sets. The Sensitivity sample set (n = 51) was evaluated only for TP and FN only (*TN and FP not required for Sensitivity*). The Specificity sample set (n = 59) was evaluated for TN and FP (*TP and FN not required for Specificity*). Both these sample sets were merged as suggested by the Reviewer, and all samples in the combined sample set (n = 110) were evaluated for TP, TN, FP and FN to derive Sensitivity and Specificity. The FN in question was reported in a sample which was previously evaluated for TN and FP only (erstwhile Specificity set). Thus, this FN value was not reported in the prior Supplementary Table S5, but is now reported in Supplementary Table S7.

***QUERY 2: I suggest to move the four numbers used for calculating each statistic in Table 1 from the supplementary table to Table 1. This will help readers understand the underlying sample sizes, although the 95% CIs could hint on how big the underlying numbers are.***

**RESPONSE:** As the Reviewer rightfully suggests, the 95% CI values serve to convey the significance of the underlying sample (+variant) numbers. Since Table 1 is intended to provide a snapshot of the CellDx performance characteristics, incorporating numbers for FP, FN, TP and TN might render the Table complex. However, in appreciation of the point raised by the Reviewer, Table 1 Legend has been updated to explicitly mention that FP, FN, TP, TN numbers are provided in Supplementary Table S7 for benefit of interested readers.

***QUERY 3: In Supplementary Table S5, it is titled "S7". It is unclear what "(Mean depth 500x, >99.1% coverage >100X)" in the title means. After reading the manuscript multiple times, I see the authors meant they aimed at a mean depth of 500X, which was not a result data of the study. Please consider to remove from Table S5.***

**RESPONSE**: The Legend for Supplementary Table S5 has been revised to exclude the indicated text; the parameters indicated in this sentence are described in the Methods (Sequencing Data Analysis) and Results (NGS Data Analysis) Sections.
